# The feasibility of implementing food-based dietary guidelines and food graphics in Ethiopia

**DOI:** 10.1007/s12571-022-01335-3

**Published:** 2023-01-19

**Authors:** Tesfaye Hailu Bekele, Namukolo Covic, Dawit Alemayehu, Laura E. Trijsburg, Inge D. Brouwer, Edith J. M. Feskens, Jeanne H. M. de Vries

**Affiliations:** 1 Ethiopian Public Health Institute, Addis Ababa, Ethiopia; 2 Division of Human Nutrition and Health, Wageningen University and Research, Wageningen, the Netherlands; 3 International Food Policy Research Institute, Addis Ababa, Ethiopia

**Keywords:** Feasibility, Dietary guidelines, Practicality, Acceptability, Understanding

## Abstract

This study aimed to test the acceptability, cultural appropriateness, consumers' understanding, and practicality of the Ethiopian food-based dietary guideline's messages, tips, and food graphics. A qualitative study design was applied with focus group discussions and key informant interviews. Four different participant groups were included: 40 consumers, 15 high-level nutrition experts, 30 frontline community health extension workers (HEWs), and 15 agriculture extension workers (AEWs) to incorporate different stakeholder perspectives. Data collection was conducted using 7 focus group discussions (FGDs) and 30 key informant interviews (KIIs). Collected data were coded and analyzed using QSR International NVivo V.11 software. Most of the study participants were highly interested in implementing the dietary guidelines once these guidelines are officially released. Based on the participants' views, most of the messages align with the current nutrition education materials implemented in the country except the messages about physical activity and alcohol intake. However, participants suggested defining technical terms such as ultra-processing, whole grain, safe and balanced diet in simpler terms for a better understanding. Practicality, affordability, availability, and access to the market were the major barriers reported for adherence to the guidelines. To be more inclusive of cultural and religious beliefs, findings show that the guideline should address fasting and traditional cooking methods. In conclusion, the dietary guidelines were well received by most stakeholders. They are thought to be feasible once feedback on wording, affordability, availability, and access is considered in the messages, tips, and graphic designs.

## Introduction

Ethiopia strives to end undernutrition and prevent the rising burden of overweight/obesity and non-communicable diseases. This is done through different policy instruments, including a multisectoral food and nutrition policy and strategy, a nutrition-sensitive agriculture strategy, and a health and agriculture transformation plan (Bossuyt, 2019; Datiko & Lindtjørn, 2009). These policies and programs include actions to improve healthy lifestyles and availability, accessibility, and consumption of healthy diets (Baye et al., 2019). One priority action was developing, testing, and implementing food-based dietary guidelines (FBDG).

FBDG has been described as 'consistent and easily understandable translations of population nutrient goals into simple public messages, encouraging healthy habitual food choices to improve public health' (FAO & WHO, 1998). The primary goal of the FBDG is to encourage consumers to make healthy food choices to reduce the risk of chronic diseases and malnutrition. FBDG can also be used to inform actions to improve the food system, including practices by food industries to promote better consumption patterns. According to the United Nations Food and Agriculture Organization (FAO & WHO), national FBDG should define context-specific sustainable healthy diets by considering the social, cultural, economic, ecological, and environmental circumstances. Under current practices, FBDG develops technical guidelines accompanied by public messages and visual food graphics to facilitate consumers' understanding (EFSA, 2019).

The development of the FBDG is a lengthy evidence-informed stepwise process (Albert et al., 2007; Bekele et al., 2019). Technical guidelines need to be translated into public practical education messages and materials that the consumers can understand, remember, accept, and apply. It is important to investigate how well the dietary guidelines and illustrations are understood, well accepted and whether they are culturally appropriate and practical to consumers (Brown et al., 2011). As a result of such studies, FBDG will be more context-specific and effective in improving knowledge, attitude, and practice toward a healthier diet during implementation (Penelope Love et al., 2001). Research by Love (2002) showed that nutrition education tools, such as FBDG and visual food guides, are often misunderstood or not used appropriately by consumers (Valmai, 2002). The FAO and WHO have recommended consumer testing in the development of FBDG (FAO & WHO, 1998) and the involvement of experts and program professionals to ensure the messages are correct and relevant to the public. Furthermore, the FBDG should be understandable and acceptable by the frontline workers who implement the guidelines to the public. Across different countries, several testing studies of FBDG for specific target groups have already been conducted (Albert et al., 2007; Britten et al., 2006; Gabe & Jaime, 2019; P Love et al., 2008; Napier et al., 2018; Rohrs, 2019). The Ethiopian FBDG will be presented for the first time in this study, considering its feasibility. Similar studies during FBDG development benefit especially countries with diverse cultures, differences in socioeconomic conditions and dietary pattern by contextualizing the guideline for effective implementations.

**Fig. 1:**
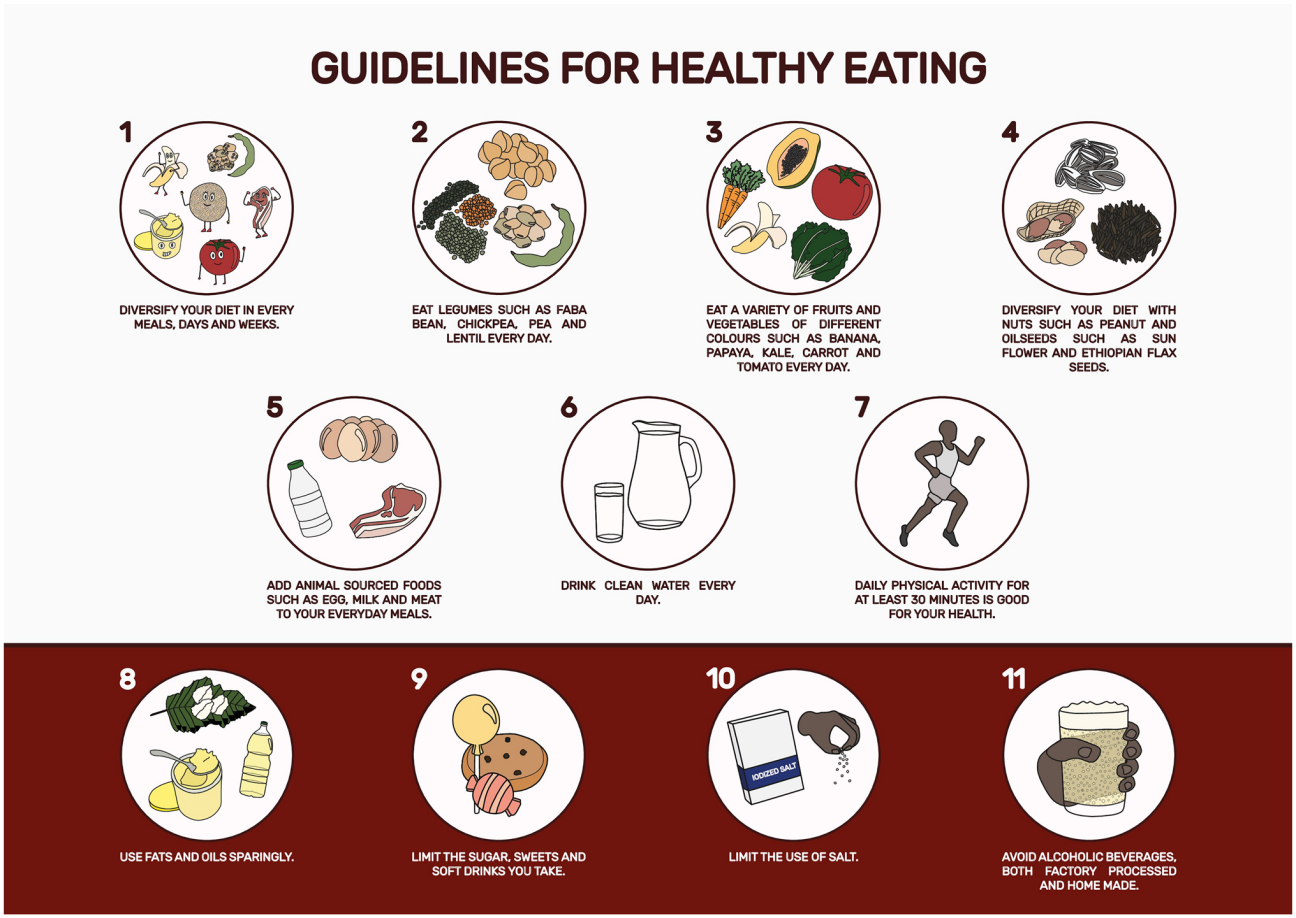
The 11 public messages for the Ethiopian Food-Based Dietary Guidelines

This research aimed to test the acceptability, cultural appropriateness, consumer understanding, and practicality of Ethiopian FBDG, which Ethiopian Public Health Institute (EPHI) has recently developed in collaboration with FAO and Wageningen University. Eleven key public messages for Ethiopian FBDG and tips and food graphics were tested for acceptability, cultural appropriateness, understanding, and practicality. Out of the 11 public messages, the first 7 encourage consumers to implement healthier dietary practices habitually, whereas the last 4 public messages advise limiting certain food groups to stay healthy (Fig. 1). The detailed food guide, including the tips for every message and food graphics that characterize different food groups, is supplementary to this paper.

## Method

The approach for investigating the feasibility study for Ethiopian FBDG was according to a proposed design by Bekele et al.(Bekele et al., 2019). The first component (acceptability) concerns to what extent FBDG and food guides motivate consumers to change their behavior (Goldberg & Sliwa, 2011). The second component (cultural appropriateness) examines various factors about how well the guidelines fit with current customs (FAO & WHO, 1998), and food habits. Guidelines that propose radical changes from the current diet or undermine religious or cultural beliefs will not be appropriate (Anderson & Zlotkin, 2000). The third component (understanding) tests whether FBDG is written in an understandable manner (Anderson & Zlotkin, 2000), considering population literacy. The terminology should be simple, self-evident, and refer to foods instead of nutrients. The general population should readily understand visual representations of the FBDG and be able to easily remember them. The fourth component (practicality) recognizes the social, economic, agricultural, and environmental conditions associated with the foods and eating patterns (Anderson & Zlotkin, 2000). Considering the geographical variation, the recommended food groups should be widely available and accessible to most people.

### Participants and setting

To incorporate different stakeholder perspectives and involved interests, four participant groups were included on purpose: high-level nutrition experts, frontline community health extension workers (HEWs), frontline community agriculture extension workers (AEWs), and consumers in a selected rural setting of Amhara regional state and urban setting of the capital city, Addis Ababa. The high-level nutrition experts (15) were selected based on their role in nutrition leadership and experience in developing communication materials for nutrition behavior change in Ethiopia. Their nutrition leadership skills and experience included multisectoral nutrition program coordination, policy-making, and enforcement. The public messages and graphics for the Ethiopian FBDG and the data collection tools to test the feasibility were revised based on the input from these high-level nutrition experts. This step is considered a pre-test for the data collection questionnaires and a final revision of the guidelines before being tested at the community level. These are the country experts who can give technical input for the work. Their overall collected data were included in the analysis of the other dataset for further analysis.

A total of 30 HEWs (15 from Amhara and 15 from Addis Ababa) and 15 AEWs (from Amhara) participated in this study. Field supervisors and health and agriculture office heads selected these health and agriculture extension workers randomly from available lists in selected woredas (districts). The HEWs and AEWs were included in this study because of their role in delivering nutrition behavior change communication, health extension packages, and agriculture extension packages at the community level (Berhane et al., 2018a; Teklehaimanot et al., 2007).

Consumers were represented by women of childbearing age (15–49 years). This choice was made because of the predominant role of mothers as the ones in the household responsible for purchasing, preparing, and allocating the food for consumption (Berhane et al., 2018b). Furthermore, HEWs and AEWs target women for nutrition intervention programs and behavioral change communications. A total of 40 women (15 from Addis Ababa and 25 women Amhara regions) participated. To be included, women needed to be between 15–49 years old, married and living with their families in urban or rural settings. Individuals were excluded from the study if they were a household member working in the health care, nutrition, or fitness industry, on a medically prescribed diet, or considered a nutrition expert. The women were recruited in collaboration with health extension workers using purposive sampling.

### Study design and data collection

A qualitative study design was applied in this study. Data were collected through focus group discussions (FGDs) and key informant interviews (KIIs) using structured discussion topics and questionnaires. An overview of the KIIs and FGDs conducted is represented in Table 1. All FGDs and KIIs were carried out in Amharic except one FGD (carried out in English) by experienced interviewers (master's degree holders in public health nutrition with experience collecting qualitative data) trained for 5 days. The dietary guidelines that are ready for evaluation were also translated into Amharic. The study focus areas, key questions, possible outcomes, and the target study population were adapted from a method on feasibility studies designed by Bowen et al. (Bekele et al., 2019; Bowen et al., 2009).

### Focus group discussions

The perspectives on the cultural appropriateness and acceptability of the FBDG were expressed through FGD. In the focus group discussions, participants were encouraged to speak freely, express their understanding and feelings about specific topics in-depth, and react or build on the opinions of other participants(Britten et al., 2006).

In total, 7 FGDs were conducted. The discussions took place at locations that were easily accessible to the participants. At every FGD, at least one moderator facilitated the discussion, and one observer was responsible for taking notes, recording the discussion, and taking photos. The sessions lasted for a range of 90–120 min (5 min of opening, 10 min of transition into the main discussion, 60–90 min of the main discussion, 10 min of summary, and 5 min of acknowledging the participants) and had a range of 10–12 participants per group. All sessions were recorded with permission.

### Key informant interviews

Key informants were selected based on their skills, position within society, or ability to provide a deeper insight into the research topic. The key informant interview (KII) questions were loosely structured, allowing a free flow of information (Marshal, 1996). The goal was to get insight into whether the FBDG were understood and practiced to translate into daily food choices. The initial KII questionnaire was developed based on a literature review about the understanding and practicality of the FBDG. The draft questionnaire was pre-tested in a household, that was not part of the study, and subsequently fine-tuned.

In total, 30 key informant interviews were conducted; 5 among high-level nutrition experts, 10 HEWs, 5 AEGs, and 5 women (Table 1).

**Table 1:** Overview of Focus Group Discussions and Key Informant Interviews conducted

	WCA in an urban area	WCA in a rural area	Nutritional Experts	HEWs in urban and rural	AEWs in rural	Total
FGDs (number of participants)	1 (n = 10)	2 (n = 20)	1 (n = 10)	2 (n = 20)	1 (n = 10)	7 (n = 70)
KIIs number of participants	n = 5	n = 5	n = 5	n = 10	n = 5	n = 30
Total by subgroup	40 WCA		15 experts	45 extension workers		100

*WCA* women of childbearing age, *HEWs* health extension workers, *AEWs* agriculture extension workers, *FDGs* focus group discussions, *KIIs* key informant interviews

At each interview, the enumerators gave key informants the food guide, which included key messages, tips, and food graphics, to read for 10–15 min. The enumerators then spent 5 min reading the material to introduce the messages to the study participants. The data collectors read the messages and tips for consumers who could not read them and showed them the dietary guidelines and graphics. After data collection, the saturation level was checked for both FGDs and KIIs by comparing the degree of repetition in the new data to the previous data to ensure that the sample size was adequate.

### Data analysis

The KIIs and FGDs were transcribed and translated from Amharic into English. All transcriptions and translations were checked and adjusted by the two research coordinators. Data analysis was done using QSR International's NVivo V.11 software. Both FGDs and KIIs were analyzed together since the information complemented each other and showed the complete picture of the four components (acceptability, cultural appropriateness, understanding, and practicality). After the initial series of component mind mapping, defining, and refining, the dominant themes and sub-themes were discussed within the research team until consensus was attained. Responses were coded under these themes and sub-themes to synthesize the information.

## Results

The findings from the FGD and KII are presented in four main sections: acceptability, cultural appropriateness, understanding, and practicality. The four main sections are subdivided into high-level nutrition experts, health and agriculture extension workers, and women of childbearing.

### Acceptability of the Ethiopian food-based dietary guidelines

**Fig. 2:**
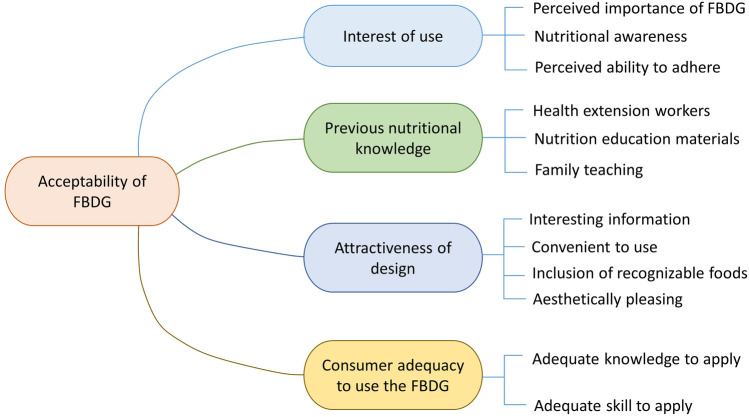
Mind map of responses given on the acceptability of the Ethiopian FBDG

The acceptability of the FBDG was determined by the attractiveness of the graphics design, consistency with other nutrition messages, interest in using the food guide, and consumer adequacy to use the FBDG (Fig. 2). According to the summary from this section, most of the participants in this study from implementers (high-level nutrition expert and community workers) and end users (women) expressed an eagerness to put the dietary guidelines into practice once they were officially released. Most of the messages in the dietary guidelines were considered to be consistent with current communication materials for nutrition behavior change. There is a need for more practical examples of physical activities and cooking demonstrations. According to the participants, the current version should be revised to make it more user-friendly to implementers and end-users. The detailed study participant-suggested revisions are broken down into sub-sections below.

#### High-level nutrition experts

All participants in this category mentioned that they are interested in using the new FBDG in their professional work once it is completed and officially released for implementation. There are a variety of nutrition education materials available that are not harmonized for a common set of messages and are targeted specifically at mothers or children. The nutrition expert group agreed it was worth having one standard national food-based dietary guideline as a reference.

*"I'm excited to see this initiative because I haven't had any country-specific dietary guidelines to discuss with my students for many years when teaching about a healthy diet or dietary diversity at the university. I was mostly referring to the dietary guidelines of Canada, the United Kingdom, or South Africa, which are in a completely different situation than Ethiopia. Because our country's nutrition problem is so severe, I'm very interested in advising the general public based on this guideline once it's completed." –* (High-level nutrition expert, ID42)

For each food group, mascot images with a human character are used in the dietary guidelines' graphics design. Some nutrition experts thought the graphics were appropriate for both children and adults, while others thought they were only appropriate for children because the food representation of human characters deviates from the food's natural look, which adults may not find appropriate for communicating a healthy diet.

*"I think the idea of the pictures with faces is very attractive and makes it more interesting. It's less text… more illustration, fun faces, bright colors. You know the fine-tuning of the illustrations and changing some of them based on the feedback and revising the messages needs to be considered"*– (High-level nutrition expert, ID43)

Most of the nutrition experts mentioned that the messages in the food-based dietary guidelines were in line with the country's current food and nutrition policy and strategic plan. Most dietary guideline messages are used in the current nutrition behavioral change communication materials. There are new messages in the FBDG, such as encouraging physical activity and limiting sugar and fat consumption. Furthermore, the balanced diet or diet diversity advice, which suggests eating at least 4 out of 6 food groups every day, suggested being checked against other countries and WHO recommendations and whether the minimum energy and nutrient requirements can be met with this advice. Most respondents stated that the physical activity messages require more explanation with practical examples, considering the various target populations for implementation. The urban and rural communities have different lifestyles and work environments and therefore need targeted advice on types of physical activities, intensity, and duration.

#### Health and agriculture extension workers

The health and agriculture extension workers believe that this guideline is relevant to the community. Based on their view, it is well aligned with the existing nutrition education materials and has additional information that will help to prevent non-communicable diseases through a healthy diet. However, they also stated that these dietary recommendations, tips, and graphics illustrations do not consider pregnant or breastfeeding women. They stated that because they are part of the population above two years, it is critical to incorporate a message specifically for them.

*"I missed out on demonstration on food preparation and recipes for pregnant women. I want mothers to have a proper diet to have a healthy baby during their pregnancy period. I do not want to see a generation affected by stunting."–* (Health extension worker, ID60)

According to health extension workers, nutrition awareness is still lacking at the community level, and the level of awareness varies by location and level of education. They also believe that following this guideline will help them better understand a healthy diet to teach their community. Some participants suggested that some of the messages need a detailed explanation and more practical examples to implement at the household level. Other participants did not agree with this idea.

*"Cooking skills require knowledge, understanding, and skill! This has to be taken into consideration in the guideline. The skill itself is the main one! For example, skill is a matter of preparing the food in the right way with adequate knowledge. If we say we are cooking the food, while the food stays on fire for a long time, the nutrient content will be lost."* – (Health extension worker, ID64)

#### Women of childbearing age (consumer) both in the urban and rural setting

Both rural and urban women agreed that having a dietary guideline is important for healthy eating and preventing malnutrition and related disease. The dietary guideline was seen as useful by women because it can teach them why certain foods are healthy. The majority also stated that the dietary guideline would encourage them to include the listed foods in their diets while also informing them about their importance.

"*As I have seen it today, I understand that peas and beans are protein, and it is good for us. We see pineapple in Debre Birhan, but we don't know its benefits. But when we look at it, and what we see here in the pictures, I am now looking and realizing that it is healthy and suitable for us. What I am realizing and understanding now is that it has gone from our community, and we see it comes back as very useful*". – (Rural woman, ID15)

The dietary guideline was mostly in line with what the women had previously heard from health extension workers. In all three FGDs, the women did recognize the nutritional information stated in the food guide, and they said that much of the information was not new to them. The urban women, however, mentioned some contradictions. The message about not overcooking vegetables ran counter to previous advice, which stated that foods should be thoroughly cooked. In addition, one of the women seemed to find the message about eating fats and oils in moderation to be contradictory. She claims she was told that 'fat things' should not be used.

According to rural and urban women, the dietary guideline is convenient and interesting. The main reasons given are the presented variety of familiar foods and different options from the food group. The women were enthusiastic about the aesthetics of the guide and expressed with words like beautiful, satisfying, and appealing. According to a rural woman, the dietary guideline appeals to her because it includes a list of alternatives, such as the different kinds of fruits and vegetables. One of the urban women remarked that the first page of the dietary guideline is uninteresting and not attractive to those with less money to spend.

According to the responses of many of the women, awareness of the causative relationship between nutrition and health is necessary for these guidelines to be used by the community. If this awareness is not there, the intrinsic motivation to apply such guidelines will be lacking.

*"Most of the time children, babies would use it (sugary foods), and sometimes mothers do not prevent them from using it. They would use candy a lot. It's a matter of awareness".* – (Urban participant, ID21)

Lack of practical knowledge about implementation of healthy diets might reduce the acceptability of FBDG. Focusing on what is feasible when it comes to healthy eating was suggested in resource-constrained situations to make it simpler to apply the recommendations by providing affordable food choice.

### Cultural appropriateness of the Ethiopian food-based dietary guidelines

**Fig. 3:**
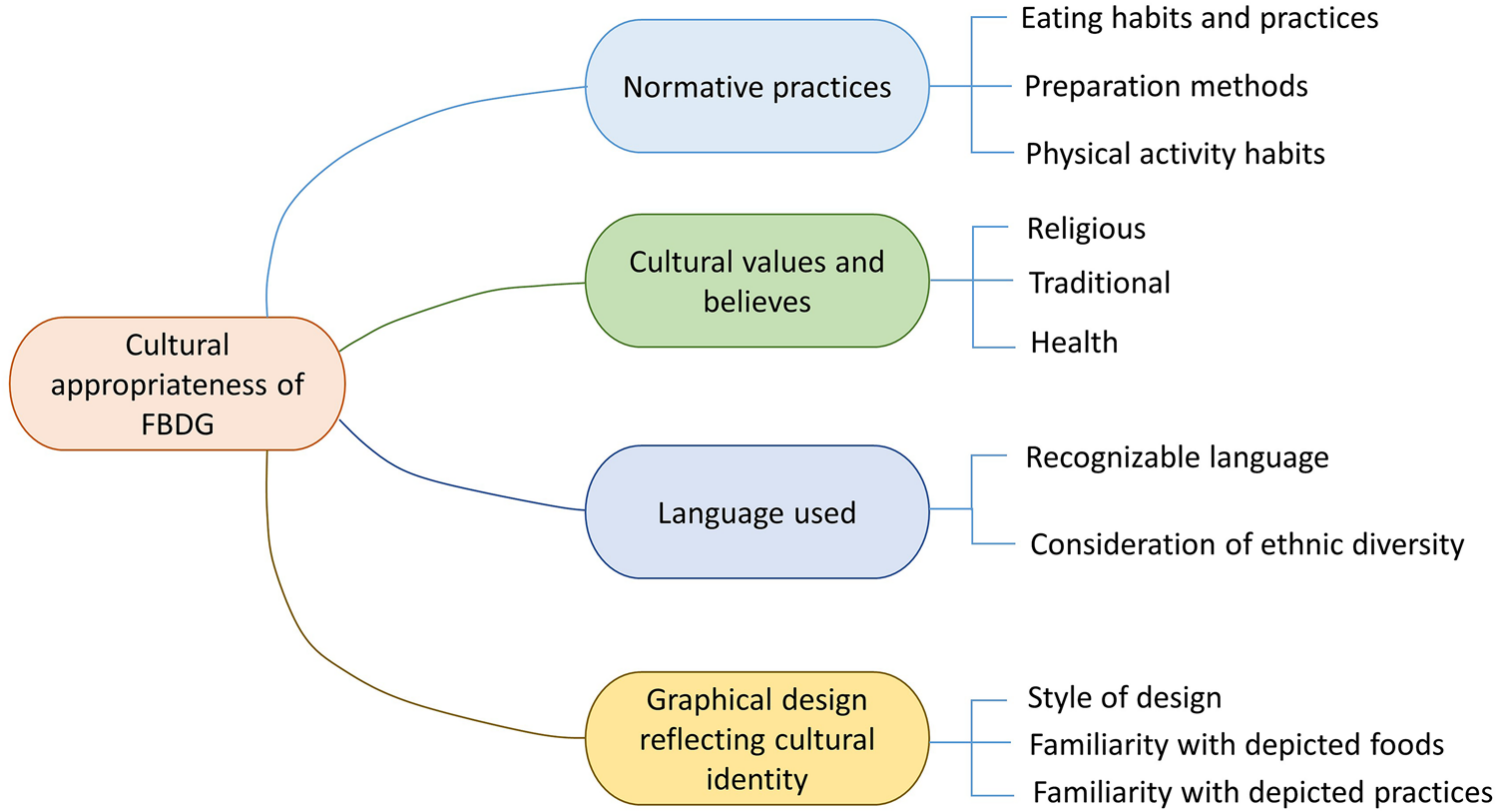
Mind map of responses given on the cultural appropriateness of the Ethiopian FBDG

The cultural appropriateness of the FBDG was determined by the normative practices, cultural values, beliefs, language used, and graphical design reflecting the cultural identity (Fig. 3). The summary from this section indicated that Ethiopians are accustomed to fasting, and animal-source foods are prohibited during the fasting period in the Ethiopian Orthodox Church. The dietary guidelines include recommendations on cooking healthy, avoiding overcooking, not pouring water after boiling vegetables, and limiting the amount of salt and oil used. Dietary guidelines consider cultural values and beliefs to promote a healthy diet. The cultural appropriateness is detailed into three sub-sections below.

#### High-level nutrition experts

The majority of experts' feedback indicated that it is critical to consider Ethiopia's fasting tradition and foods that are allowed or prohibited due to religious practices. According to the nutrition experts, dietary guidelines will be more culturally appropriate if they are translated into various local languages. The way graphics are designed and messages translated into Amharic, has to be more culturally suitable for various population groups according to some participants.

The nutrition experts group highlighted that food safety has to be communicated to the public to handle food in a safe way. They also mentioned that it is relevant to integrate food safety messages like accessing safe and clean water and keeping the foods safe as part of traditional food preparation. According to the participants in the study, it is more appropriate to improve overcooking skill by avoiding overcooking and limiting foods risk factors for diseases.

*"The message about legume consumption tells me, I have to consume legumes in every meal every day. However, it does not say much about in what form I can consume such as roasted, boiled, Shiro(stew) form, germinated or boiled/roasted..."* – (High-level nutrition expert, ID40)

According to a few experts, it is critical to promote wild fruits and vegetables as part of traditional diets in Ethiopia. Besides, adapting food lists based on agroecological zones and availability will benefit the country's diverse communities.

#### Health and agriculture extension workers

According to some health extension workers, certain foods are prohibited by the community without any scientific evidence. This guideline has to change those beliefs by mentioning the correct way of consuming different foods to have the right information.

*"The community doesn't allow cabbage to be eaten by pregnant women because of parasites. Normally, a pregnant woman can't take medicine in early pregnancy. The message gives information on how pregnant women can consume vegetables and fruits."*- (Health extension worker, ID59)

Most agriculture and health extension workers mentioned that Ethiopia has diverse ethnic groups and a larger population who do not speak the national language. Many households, restaurants, and hotels serve high fat and oil content food. According to one of the respondents, there should be a clear message to change this cooking tradition. It is critical to translate this guideline into various local languages, at the very least Oromiffa and Tigrigna.

#### Women of childbearing age (consumers) both in the urban and rural setting

The majority of the women said that the food guidelines reflect the traditional Ethiopian diet, full of fresh products and do not include many packaged and foreign foods. Some rural women did mention some products not represented in the dietary guideline yet were often used by them. These are coffee, tea, and honey, but it was unclear what the nutritional advice was concerning these traditional Ethiopian food products.

"*Coffee, for example, is now required to be included as one of the foods/drinks? There's coffee, but where does it fit? There is no mention of it. Do we put it in the grain category, or do you mean inside the drink? We had no idea which category it belonged to*". – (Rural woman, ID30)

Most of the women believe the dietary guideline does fit their religious beliefs as they are easy to adapt to the different religions in Ethiopia. Women who consider themselves orthodox-Christians and practice fasting also believed the dietary guideline fits their faith well. The main reason for this was the separation of the fasting foods from the non-fasting foods within the guidelines.

Some women expressed their belief that these food guides only needed to be followed when someone was not in good health. It was not clear to them that the guidelines function as prevention measures and therefore help them prevent certain diseases. Most women believed that processed and packaged foods were inherently unhealthy. They tended to think that homemade and fresh foods and beverages were inherently healthy or not harmful.

Participants unfamiliar with all the foods shown in the dietary guideline did not see it as a problem. The language used within the dietary guideline was familiar or not disrupting to them, apart from the foods they had not been familiar with before. They were aware that different foods are produced within other areas and communities in Ethiopia, and different eating habits are dominant.

Although the women were generally positive about the overall design, some were issues with how the illustrations were designed. Those women believed the graphics illustrations were not designed based on their culture as they were designed too modern. This modern graphics design makes the guide mostly suitable for those who can read.

"*... The diagrammatic illustrations look modern and should be made according to our culture. I mean that these foods are ours, but the characters are modern. This dietary guide is prepared for the community to understand it by reading rather than observing its diagrammatic illustrations.*"- (Rural woman, ID37)

Salt should be added after cooking rather than during cooking, which is not how most participants prepare their food to retain iodine. According to a participant, it is more typical to use salt when cooking.

### Understanding the Ethiopian food-based dietary guidelines

**Fig. 4:**
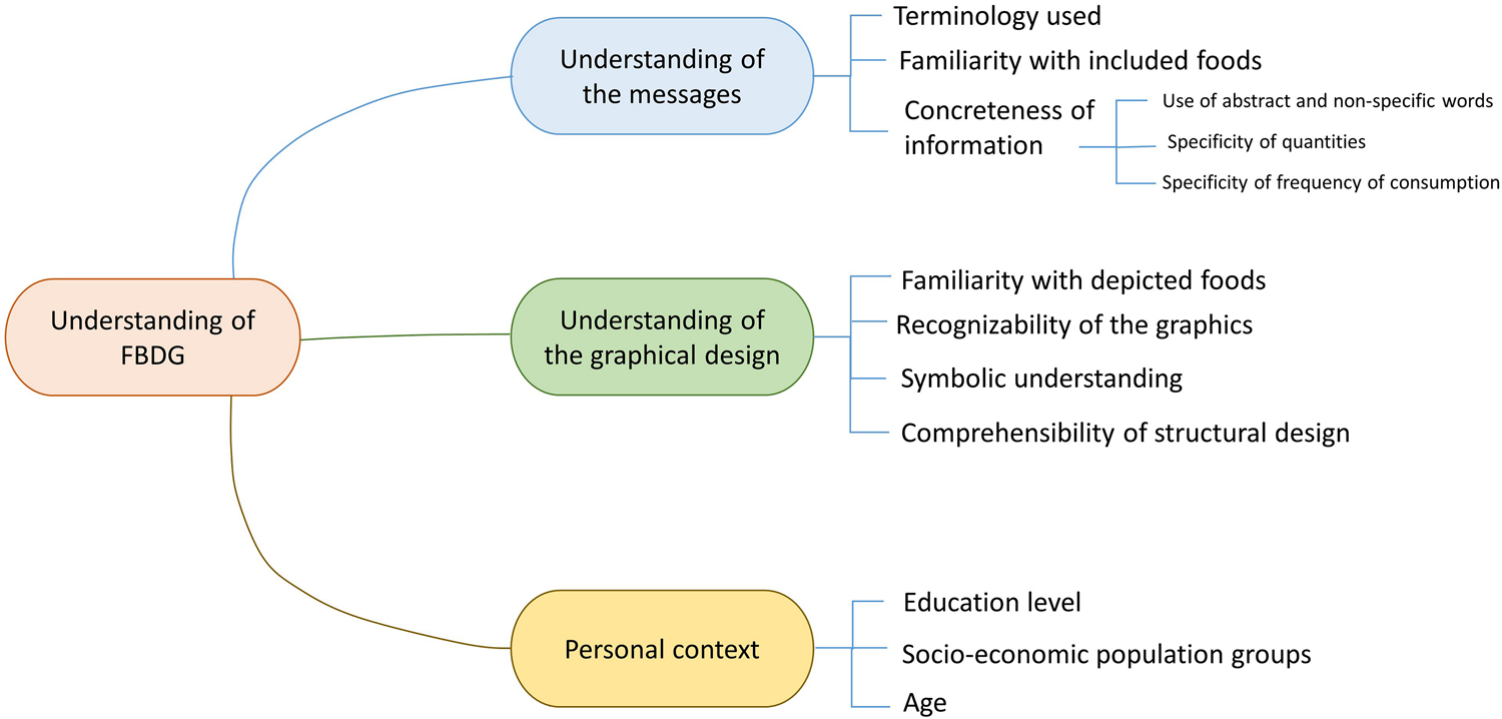
Mind map of responses given on the understanding of the Ethiopian FBDG

The understanding of FBDG covers personal understanding of the messages, food graphics, and unique context (Fig. 4). The technical terms used in the dietary guidelines, such as balanced diet, ultra-processed, whole grain, and safety, require a simple definition. Since half of the adult population in rural areas cannot read or write, the graphics illustration must be self-explanatory and use a familiar image–the detail described in three sub-sections below.

#### High-level nutrition experts

A majority of the respondents believed that the 11 public messages were comprehensive. Still, the illustration used in the public messages and the tips and the food graphics combined for every message is not clear enough. According to the majority of respondents, some of the terminologies, words, and phrases used for illustration may change the interpretation of the main message of the guideline. In addition, some of the technical terms and phrases used in the messages, such as diet diversify whole grain, sparingly, limit, safe, clean, and ultra-processing, need a simplified definition.

*"Only experts in the field know what the term ultra-processing means. It's good to find a simple description of ultra-processing.....like removing the upper cover of cereals. In Amharic, we call it 'Yetefetege' meaning removing the cover for cereals and 'Yetekeka' meaning split for legumes."* – (High-level nutrition expert, ID51)

In addition to Amharic, the respondents suggested that the final food-based dietary guidelines be translated from English to other local languages. According to the experts, the most important aspects that require extra attention during translation are sentence structure and word choice. The color and shape of the foods in the graphics are not similar to those of natural foods. Half of the Ethiopian population in rural areas cannot read or write; graphic illustrations are important to recognize without text. None of the graphics indicate food safety. Incorporating food safety advice in the right messages is important, especially for fruit and vegetable consumption and food preparation and consumption. The packages used for salt, milk, yogurt, and the serving utensils for water and cheese are unfamiliar in Ethiopia.

Nutrition experts also mentioned that users might be confused by how the circle resembles a plate is used for each graphic illustration of the 11 messages. They suggested the graphics in the dietary guideline might be easier to understand if we use the traditional plate (mesob) to represent both the urban and rural communities. The second sentence at the top of the dietary guideline says that "foods that are not mentioned in the graphics can be eaten sparingly," which is not specific enough to understand what type of foods and the portion size be consumed.

*"...I am a bit lost about how to apply these messages. The foods I will eat in every meal every day are too much. I am not sure if I'll eat legumes every day; it seems boring. Eating meat every day doesn't seem realistic. There should be a way to use alternatively with portion size..."* – (High-level nutrition expert, ID40)

Most experts stated that the quantity of every food or food group that needs to be consumed every meal or day has to be incorporated in the final messages. They also mentioned there should also be a minimum possible portion size stated for the message that limits intake. The portion size has to be noted to understand and implement it easily. The guideline about water intake in message 6 is more focused on quality than quantity. However, the quality and quantity of drinking water are equally important in Ethiopia to mitigate water-borne disease, malnutrition, and non-communicable diseases. The alcohol message also needs clarification. The last message of the guideline says to avoid alcohol consumption, whereas, in the illustration, it recommends not more than one glass per day. It is better to be consistent with the message about alcohol intake to avoid confusion.

#### Health and agriculture extension workers

Some extension workers stated that consuming a diverse range of foods daily may be difficult unless the simplest method is described in detail, along with the appropriate amount and cost. Ethiopians prepare fermented bread (enjera) and serve it with other food groups. They also mentioned that explaining a healthy diet or a varied diet using enjera, a common Ethiopian dietary habit, might be simple.

*"We, health extension workers, should increase the community's awareness about a balanced diet by educating them to work with the available foods such as cabbage, carrot, potato, legumes, cereals, and animal source foods so that people can eat healthier. We should do it by considering what is available in the community."* – (Health extension worker, ID46)

Certain foods such as anchote, cassava, and grass pea were unfamiliar to most participants in the two regions. Extension workers also indicated that graphics need a major improvement so that people from the rural who cannot read the text can easily understand the advice with minimum support. The message on drinking clean water every day is important for health. Water-borne diseases are one of the major causes of malnutrition in Ethiopia. Therefore, more detail should be on how people clean drinking water if their drinking water sources are not clean enough.

*"There are different treatment methods for drinking water like filtration, chemical (aqua tap), and boiling. When people keep drinking water, it should be covered in a container narrow at the top. Water has to be kept and served in a clean environment so that people can prevent water-borne disease such as diarrhea."* – (Health extension worker, ID49)

According to extension workers, limiting salt and sugar in our diet is a message that is new in this guideline. However, normally people know about it from the media. However, it is not incorporated in the previous nutrition education materials. Normally, salt intake is high, and people also consume more sugar from time to time.

#### Women of childbearing age (consumers) both in the urban and rural setting

Not all participants were familiar with the foods mentioned in the food guide, which hindered understanding. The most commonly unfamiliar foods were anchote, cassava, and kocho (or false banana). Only some rural women seemed to be unfamiliar with melon and peanuts. All participants were unfamiliar with the term 'processed meat' regarding the tips on message 5: avoid processed meat regularly. Many women explained the importance of being physically active with responses, including 'It relaxes the body, helps with digestion, or protects against diseases'. Yet, how to putting it into practice was not clear to all women.

*"It is about moving my hands and feet for 30 minutes. Sport is not just about standing and running; it is also sport when I work at home. Moving up and down to work in the house is also an exercise. So, it is not a difficult task unless you are not aware of it. But there may be an awareness problem*". – (Urban woman, ID21)

Most of them also explained diverse eating as consuming different types of foods through the timeslot in which these foods should be eaten unambiguous. Some women stated that eating different foods for breakfast, lunch, and dinner every week is important. In contrast, others indicated that eating different foods for breakfast, lunch, and dinner every day is important.

*"Diversifying or balancing means vegetables may not be available daily, but we can get vegetables weekly; if we buy bananas, we can make it four times or more for a family. Then, papaya for another day since it does not stay longer once cultivated. Although we don't get it every day, we can serve vegetables at our weekly meals."* – (Urban woman, ID16)

Several women did not understand the recommended frequency of consuming certain food groups. In the context of the dietary guideline tips, the Amharic translated the word "sparingly" was interpreted by the participants as "processing, cleaning, and preparing your food hygienically and carefully" (Foods that do not appear in the food graphic can be eaten sparingly).

All women seemed to understand the importance of limiting the use of salt. Terms used to explain their salt consumption are "in moderation, proper usage or limitation." All women seemed to be well aware of the health consequences of eating excess sugar. Most women understand that the FBDG recommended not drinking alcohol; others believed the message was about moderate or occasional consumption. The women had a different understanding of how often water should be consumed. One talked about drinking water every hour, whereas another woman said 'always' drinking water was unhealthy and indicated the presence of disease.

The dietary guideline mentions eating legumes instead of animal products on days of fasting. Yet, when asking about the food to replace meat, legumes were not often the first food mentioned to be eaten. Most women, especially rural women, said they eat certain vegetables instead. Urban women sometimes mentioned legumes.

### The practicality of the Ethiopian food-based dietary guidelines

**Fig. 5:**
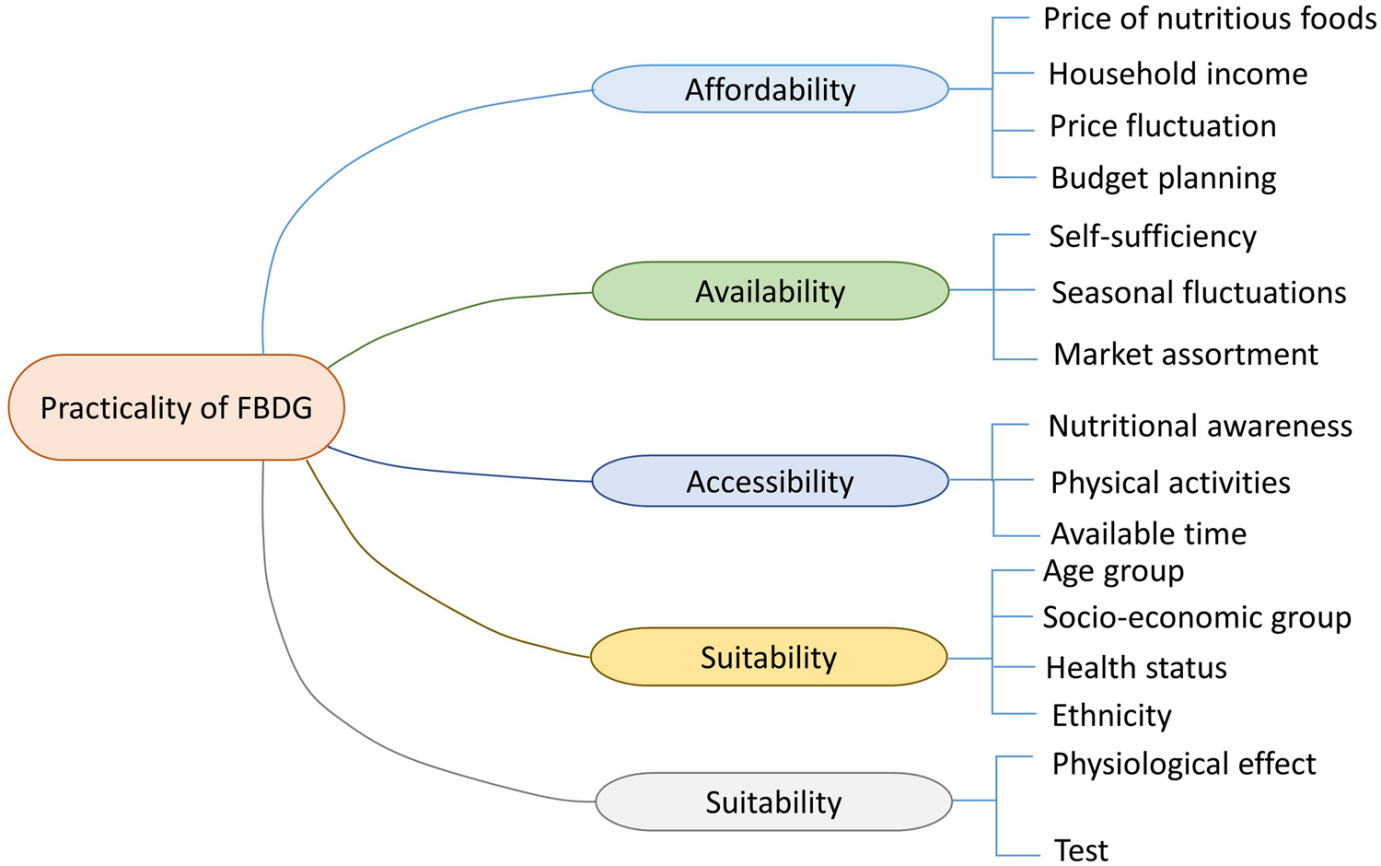
Mind map of responses given on the practicality of the Ethiopian FBDG

The practicality covers affordability, availability, accessibility, suitability, and favorability of the FBDG (Fig. 5). In both urban and rural areas, affordability has become a major concern. The long-distance between home and market is seen as a major impediment to putting the guidelines into practice daily. Seasonality and low productivity can limit the availability of various fruits and vegetables. More efforts should be made to improve the agricultural production of those fruits and vegetables. To enhance low micronutrient intake, fortified products, improved seeds like orange flesh sweet potato, orange maize, and biofortified legumes should be promoted. Physical activity and limiting added salt and oil during food preparation require a more detailed explanation and consideration to change current practice.

#### High-level nutrition experts

According to the respondents, some of the key determinants of practicality to implement the food-based dietary guidelines are price, availability, accessibility, seasonality, religion, and culture. They also indicated that these guidelines' practicality in the urban areas would differ from rural areas. According to them, the urban population will have relatively good access to different foods. However, most low-income people cannot afford some of the food groups. The low-income rural community will have both accessibility and affordability as a challenge. The rural middle and high-income groups will have limited access to healthier diets.

"*...this guideline says eat or drink animal source foods every day. We know how expensive animal source foods are at the moment. Instead of saying eat or drink animal source foods, It's better to say add animal source foods to your daily diet.*" – (High-level nutrition expert, ID44)

According to the experts, legume consumption is practical and familiar to the community compared to animal-sourced foods, fruits, and vegetables. They also mentioned physical activity is difficult to communicate for different living conditions in urban and rural settings. Alcohol intake is part of the tradition in Ethiopia on different occasions. They suggested that limiting their intake to 1 or 2 glasses per day is a more practical advice. It might be impractical for the public to avoid alcohol intake.

*"...Most of the time, alcohol is consumed out of working hours and consumed the time that should have been spent with family. The money used for alcohol intake costs to be shared with other family members. By explaining those disadvantages, it's possible to bring a behavioral change and minimize the intake."* – (High-level nutrition expert, ID48)

The experts thought the practicality of these guidelines would be more challenging for pastoralists who live mostly with their indigenous culture and eating habits in remote areas. They also indicated a special need for further adaptation for the FBDG for the pastoralism community. This may require an anthropological studies to understand better their eating habits and access to recommend a healthy diet to the specific context.

*"... it's good to strengthen the market linkage for better access of certain food groups such as fruits and vegetables....The market linkage can be achieved by creating cooperation of youth and farmers through credit associations"* – (High-level nutrition expert, ID51)

Some of the experts mentioned limited agricultural production and lack of nutrition knowledge could also limit the ability of the population to follow the FBDG. They suggested providing fruit and vegetable seeds every year by strengthening seed multiplication centers and encouraging researchers to produce new varieties of seeds as solutions for production improvement. They also indicated that when there is a high production of certain fruits and vegetables during specific seasons, food processing will help to reduce food loss and price fluctuations. According to the nutrition experts, it is important to include the biofortified verity of orange flesh sweet potato, orange maize, and improved verity of legumes and mung beans biofortified with zinc and iron. They also advised developing a manual for food processing technologies that can be easily applied in the country. The existence of important traditional food processing and preservation techniques is also mentioned. They suggested it is good to check their effectiveness and promote these to solve seasonality effects.

The high level nutrition experts also indicated that food safety is one of the limiting factors for consumption, especially for fruits and vegetables. They believe it is good to establish a national cold chain for fruits and vegetables, and there has to be enforcement of rules on proper handling throughout the supply chain. A message about overcooking vegetables needs to be included in the guideline while cooking vegetables is promoted. In addition, experts consider fasting a limiting factor in consuming sufficient animal source foods and water. They suggested including alternative protein sources in the diet and encouraging people to drink more water with their meals during fasting periods.

#### Health and agriculture extension workers

Most of the respondents mentioned that some of the foods such as animal source foods, fruits, and vegetables are not affordable to the majority of the low-income population. They even mostly consume a lower quantity of cereals and legumes in general. The current COVID-19 problem is also a big challenge for the low-income community. Their level of poverty is even worsening due to the pandemic.

*"...the community should know about home gardening of vegetables and fruits. This will help them eat healthily and not worry about buying at a high price."* – (Agriculture extension worker, ID71)

According to extension workers, meat is consumed more frequently and in larger quantities during the holidays. Some stated that high-income people prefer to eat meat almost every day, despite leading an unhealthy lifestyle. They reasoned that the wealthier one becomes, the more likely one will become overweight or obese in Ethiopia. Most of them indicated that most people consume fruits infrequently or do so without completely understanding their importance for health, rather because they are readily available and affordable.

Some extension workers mentioned limiting the amount of fat is easy to implement since it does not cost anything for the consumers. Few extension workers indicated that people use solid palm oil because of less cost, which is not good for their health. They mentioned that people tend to eat too much fat during the holidays. There should be a way to explain why limiting fat intake, and replacing solid oils with liquid oils is beneficial. Providing training on cooking and handling the food will help prevent food and nutrient losses. The traditional way of cooking has more fat and overcooking. Few health extension workers also mentioned that time for cooking is a challenge, especially for most urban communities.

Most of the extension workers agree with the message that not drinking alcohol requires self-control. This is possible if we can persuade the consumer with our message. Few extension workers indicated people who consume alcohol should first be aware of the risks to their health. They also said that alcohol consumption has economic and social consequences. Incorporating those three aspects is critical in persuading consumers to stop drinking.

Most of the extension workers mentioned people with disabilities, those who live in extreme poverty, those who live in a remote area, and those who do not speak Amharic might face greater challenges than the general population. They suggested this population group will require extra assistance to fully implement the guidelines.

#### Women of childbearing age (consumers) both in the urban and rural setting

The biggest practical barrier for urban and rural women was the affordability of the foods mentioned in the guidelines. Many women said they did not have the economic capacity to follow the guidelines.

"*I think this dietary guide may not be practical to our society because money is needed to implement this dietary guide. Therefore, applying all the messages listed under this dietary guide is difficult. It can be a little difficult in terms of money and the cost of living we have currently.*" – (Urban woman, ID19)

This was especially the case for the guideline stating to add animal products in everyday meals. For rural women it was less a problem adding other animal products, such as milk, eggs, or cheese, into their daily meals. This was because they often had chickens or cows that produced milk for them, of which they made their cheese and yogurt.

*"Here, we can't get meat daily because we bought it from the market. We use eggs at home because there are chickens in the house. The egg doesn't cost much. It is difficult to get meat every day, but if we have from the house, we will use it".* – (Rural woman, ID25)

Rural and urban women agreed that most foods found in the dietary guideline are available in the market. However, the market is often further away for rural women than for urban women, so it is not easy to get there. This and their constraining budget causes the rural women to be more dependent on the foods they grow and their livestock. They must go to the market to purchase other foods they do not produce themselves to have a more varied diet. Some of the women mentioned how they extend the availability of foods by growing some vegetables in their garden. According to them, this gives them easy access to certain fruits and vegetables, and at the same time, it saves them money. However, not all urban women saw this as a good option since many urban families have a rented house with no backyard.

*"... it is important to plant garden vegetables to implement this dietary guide is not functional for urban dwellers but better for rural dwellers. For example, we are here in Addis Ababa; we can't plant vegetables because we live in a rented house and don't have our backyard in general"*. – (Urban woman, ID13)

Even though most foods are available at the market, as the AEWs pointed out, their accessibility may be limited. Especially the ill, weak, disabled, and older people are less capable of adhering to the guidelines. The main reasons for this were the market's geographical distance or a lack of strength, energy, or ability to travel there. In addition, a lack of time was mentioned as a barrier to purchasing and preparing these different foods.

*"Now, every time the children go to school and return, half of us will not succeed in giving them a nutritious meal. We focus on our work, and when our children arrive, the time is already run out."* – (Rural woman, ID29)

Some women said that a certain level of knowledge was needed to apply to this food guide. Knowing how to read and prepare or combine certain foods was often mentioned as a prerequisite. Therefore, they thought the guidelines to be less accessible to illiterate, old, and homeless people. Some participants found it hard to eat or drink certain foods because of their taste. Eating dishes with a little salt or consuming more water were examples of which taste is the limiting factor in adhering to the guideline. Some participants mentioned abstaining from certain foods, which gave them physical discomfort. According to them, this was especially true with foods like beans, peas, or cabbage.

Women in the study found that their age and physical status would not be physically active. The body was also seen as a constraining factor when doing physical activity. The overall consensus was that the content of the guidelines was suitable for and adaptable to the different generations and population groups. According to the women, the nutritional needs of children, pregnant or breastfeeding women, adults, the elderly, and even those suffering from a disease such as diabetes could be met.

## Discussion

This qualitative study focused on four areas to better understand the factors influencing the feasibility of implementing Ethiopian FBDG, including acceptability, cultural appropriateness, understanding, and practicality. The majority of high-level nutrition experts, health and agriculture extension workers, and consumer representatives were enthusiastic about using the new dietary guidelines, according to the findings from the acceptability of the FBDG. Most key messages in the dietary guidelines are similar to those conveyed in existing nutrition behavior change communication materials used in Ethiopia. However, the current behavioral change messages mainly focus on vulnerable population groups; infant and young children and their mothers (Workicho et al., 2021). In comparison, the current FBDG includes the key messages, tips, and graphics for the general population above 2 years, which may address dietary risk factors in the entire population (Chaltiel et al., 2022; Schwingshackl et al., 2018). The food graphics have to be more appealing, considering Ethiopian foods' natural color, size, and shape. Unfamiliar foods, such as anchote and cassava, should be replaced with more commonly consumed foods in most regions of Ethiopia. More practical examples of physical activities and ways to improve traditional food preparation methods may improve the acceptability. High-level experts suggested changes to the wording of the messages, particularly on the key messages related to meat consumption and alcohol intake.

The study team developed the topic guide for the focus group discussions and key informant interview questionnaire after conducting an extensive literature review on the acceptability, cultural appropriateness, understanding, and practicality of FBDG. In addition, similar studies conducted in other African countries were examined for their FBDG testing methodology and components (Love, 2002; Puoane et al., 2006; Scott et al., 2009; Valmai, 2002). Advice on socially acceptable norms that determine eating behavior, such as traditional and religious celebrations and socializing, may be useful in influencing healthy food choices (Puoane et al., 2006). A feasibility study on South African food-based dietary guidelines indicated that a single FBDG is appropriate for all South Africans (Valmai, 2002). Our research suggests that it is relevant to have separate FBDG for pastoralist communities and that further feasibility studies are needed for adapting the FBDG at a regional level. Because half of the adult population in Ethiopia cannot read or write, the graphics illustrations are suggested to be self-explanatory to users (CSA & ICF, 2016). In South Africa and our study, low economic status is a limiting factor for adherence to the guideline (Scott et al., 2009). By evaluating the FBDG's feasibility using the four focus areas (acceptability, cultural appropriateness, understanding, and practicability), countries can further tailor their dietary recommendations to the context of their nation for increased adherence (Gabe & Jaime, 2019; Monteiro et al., 2022).

According to our study and other study findings, dietary guidelines reflect cultural values and beliefs (Kreuter et al., 2003). For example, our study suggested considering the most common fasting practices in Ethiopian dietary guidelines to improve Ethiopian dietary habits' nutrient adequacy. According to the Ethiopian Orthodox Church, animal-source foods should not be consumed, and the majority of people stay without food and water until 3:00 p.m. during fasting days (Edae et al., 2018; Sobania, 2022). Individuals may practice fasting in different ways, such as fasting only until lunchtime (1:00 p.m.) and not eating animal-based foods. Other people avoid all animal-source foods except fish and follow a vegan diet during fasting days (Edae et al., 2018). According to a longitudinal study conducted in Ethiopia's northern region, fasting harms maternal and child nutritional status and dietary patterns. The study recommended that existing multisectoral nutrition intervention strategies in Ethiopia sustainably include religious institutions to reduce maternal malnutrition (Desalegn et al., 2018; Kumera et al., 2018). Therefore, including religious belief, fasting in dietary guidelines will assist the general public in improving their dietary habits and moving toward a healthier diet (Desalegn et al., 2018). Furthermore, more research on current dietary patterns of Ethiopian during the fasting period among the fasting population group will assist in mitigating some of the issues mentioned.

The technical terms used in the dietary guidelines should be simplified for better comprehension. When translating the English version of the FBDG into Amharic, special care should be taken with the technical terms used. A nutrition brochure text and graphics study showed that more concrete nutrition education materials improved immediate recall of information presented after reading the materials (Clark et al., 1999). The current FBDG material lacked information on portion size, which study participants identified as a gap and suggested including the recommended amount for different food groups in the final version of FBDG. The study team decided to test the dietary guidelines without amount on purpose. Because the suggested revisions from this study may be used as input for assumptions and social acceptability constraints or scenarios for the diet modeling work to develop the recommended amount (Gabe & Jaime, 2019; Maillot et al., 2010). Describing the recommended amount using the household or local measurement units and example recipes will assist implementers and consumers in understanding and implementing the dietary guidelines (Gabe & Jaime, 2019). Aside from providing information on the type and frequency of food consumed during the day, portion sizes play a role in preventing nutrient deficiencies, undernutrition, and non-communicable diseases (Daniels et al., 2009).

The study suggested to includes tips on cooking healthily, such as not overcooking vegetables or pouring water after boiling vegetables, and limiting salt and oil. According to literature, cooking and food skills include meal patterns; food preparation methods and techniques and cooking frequency; general cooking confidence or cooking ability (with foods, method, specific meals, etc.); planning food shopping and writing lists (frequency and responsibility); cooking attitudes and enjoyment of cooking; purchasing and shopping behaviors (label reading, etc.). Cooking and food skills also include food choices, menu and meal planning behaviors (including advanced food preparation behaviors), food safety and hygiene practices and behaviors (hand-washing, thawing food waste correctly, etc.), nutrition knowledge. Health consciousness and confidence relating to choosing foods and feeding others; food budgeting; barriers to cooking and food choices (time, equipment, etc.); utilization and confidence with recipes; food practices (adding salt, etc.); food preparation complexity (specific number of ingredients, etc.); food management (ensuring food lasts adequately, etc.); and source of learning to cook are important factors to consider when it comes to cooking and food skills(McGowan et al., 2017). Besides, maintaining the cultural elements such as conviviality, culinary activities, physical activity, and adequate rest while adopting a healthy lifestyle and a healthy diet will improve adherence to the FBDG (Bach-Faig et al., 2011).

According to our study, the ability of participants to adhere to the FBDG might be influenced by affordability, availability, seasonality, and accessibility. Seasonality and low productivity can limit the availability of various fruits and vegetables (Bai et al., 2020). To combat social inequalities in nutrition and health, finding nutrient-dense, affordable, and appealing food patterns should be a top priority (Abdelmenan et al., 2020; Darmon & Drewnowski, 2015). A study indicated that home gardening and chicken roosting could help increase access to and consumption of animal-source foods, fruits, and vegetables in a marginalized population (Palar et al., 2019). To meet the demand in Ethiopia for a healthier diet, there should be a strategy to improve animal farming, innovation on improved seed varieties, crop productivity, and diversity (Baye et al., 2019). Creating market linkages improves access to and affordability of nutritious foods while increasing income for the local community by providing business opportunities. To improve low micronutrient intake at a low cost, fortified products such as fortified milk, biofortified crops such as orange flesh sweet potato, orange maize, and legumes should be promoted (Wakeel et al., 2018). A more strategic approach to the country's food system will improve the affordability, availability, and accessibility of a healthy diet (Vermeulen et al., 2020). Overall, a better understanding of the sustainable food system and the development and implementation of policies and programs will result in a more sustainable implementation of the FBDG to a healthier diet (Béné et al., 2020; Harris et al., 2022). The diet optimization (modeling the current diet to satisfy energy and nutritional requirements at the lowest possible cost) of the FBDG will take into account food availability, affordability, and fasting vs. non-fasting scenarios based on the result of this study.

Furthermore, environmental sustainability specific to the country context, such as cooking with alternative energy sources, reducing food waste, developing a seasonal calendar for seasonal fruits and vegetables, and preventing post-harvest losses, should be addressed in future research related to these dietary guidelines to avoid unintended consequences due to climate change. Most participants believed that the Ethiopian FBDGs could be feasible for implementation once feedback on illustration, appropriateness, affordability, availability, and access was incorporated into the dietary guideline and tips development and graphic designs. The study's findings will be used to revise the final FBDG's messages, graphics, and tips for better comprehension, acceptability, cultural appropriates, and practicality by sharing the findings and working closely with the FBDG committee.

## Strengths and limitations

The sample of high-level nutrition experts in this study might represent the entire country because these experts have central and regional leadership and a coordination role. Ethiopia has the second largest population in Africa, with a diverse ethnic population and a 1.1 million square kilometers land area (Department of Economic and Social Affairs, 2021). We included women of childbearing age and health and agriculture extension workers from Addis Ababa and Debre Birhan representing the urban and rural setting. However, this study sample may not represent the feasibility of the FBDG to the entire population, such as to the pastoralist community. However, our findings will contribute to revising the national FBDG. Other subpopulation groups such as males and adolescents may need to be included in a future feasibility study as their involvement in the overall implementation of the Ethiopian FBDG is crucial. In addition to women, adult males and adolescents administer income, contributing to food security and diet diversity. Consumption out of home is diversified in urban settings (Ochieng et al., 2017). Men have a crucial role in deciding how much money to spend on food and how much of a household's agricultural output should be consumed rather than sold. More testing and adaptation of the national FBDG may be necessary for pastoralists and other indigenous populations living in remote areas of the country due to their difference in lifestyle and eating habits. In addition, because of the subjective nature of qualitative research methods, researchers shape the interactions with participants and their understanding of the data during the process. Researchers may not always reach the same conclusions (Pilnick & Swift, 2011).

In low- and middle-income countries, availability dietary datasets and intervention studies are quite limited to explain FBDG feasibility, our study may serve as a good example of how consumers and implementers can be involved in the development and evaluation of FBDG (Wijesinha-Bettoni et al., 2021). It is crucial to perform similar studies in various population groups and nutrition stakeholders to better implement the FBDG especially in Africa and South East Asia, where there is a widening gap between the rich and the poor, a large different in education level, inequalities in agriculture, health and nutrition outcomes (Bell et al., 2021).
